# Temporary closure of colostomy with suture before colostomy takedown improves the postoperative outcomes

**DOI:** 10.1007/s00384-017-2934-1

**Published:** 2017-11-22

**Authors:** Wan-Hsiang Hu, Ko-Chao Lee, Kai-Lung Tsai, Hong-Hwa Chen

**Affiliations:** 1 Department of Colorectal Surgery, Kaohsiung Chang Gung Memorial Hospital and Chang Gung University College of Medicine, 123, Ta-Pei Rd., Niao-Sung District, Kaohsiung City, 83301 Taiwan; 2 Graduate Institute of Clinical Medical Science, College of Medicine, Chang Gung University, Kaohsiung, Taiwan

**Keywords:** Colostomy, Operative time, Takedown, Wound infection

## Abstract

**Purpose:**

Temporary loop colostomy is a common surgical procedure used to avoid complications in high-risk distal anastomosis as well as pelvic inflammation. Issues regarding postoperative outcomes of colostomy takedown have been widely discussed in the literature, wound infection especially. Temporary closure of colostomy with suture before takedown was adopted in our study, which provided excellent traction to aid mobilization of stomy and avoided stool spillage to downgrade the wound classification to “clean contamination.” We aimed to determine the effects of the procedure on postoperative outcomes.

**Methods:**

This was a prospective case-control study at a single tertiary medical center. Patients presenting for elective colostomy takedown were included. Allis clamp (*n* = 50) or silk suture (*n* = 60) was applied to mobilize the colostomy, and results were compared. Operative time and wound infection rate were measured as primary postoperative outcomes. Univariate and multivariate analyses were used to demonstrate the association between the two groups and outcomes.

**Results:**

In univariate analyses, significantly shorter operative time (median = 57 min, *p* = 0.003) and lower postoperative wound infection rate (3%, *p* = 0.03) were noted in the group receiving silk suture. Multivariate analyses results showed that silk suture was significantly associated with both operative time (*B* = − 8.5, *p* = 0.01) and wound infection (odds ratio = 0.18, *p* = 0.04).

**Conclusion:**

With the advantage of enhancing traction and decreasing contamination, the temporary closure of colostomy with suture improved takedown outcomes, including a shorter operative time and lower wound infection rate.

## Introduction

To perform a temporary diverting colostomy is a method accepted to avoid stool from the distal bowel with anastomosis, inflammation, and trauma [1]. It is also recommended for rectal cancer patients after low-anterior resection to decrease the anastomotic leakage and reoperation rate [2–5]. Different complications were reported for patients receiving loop colostomy and ileostomy, which included ileus, dehydration, parastomal hernia, and surgical site infection [6–8].

Surgical site infection is the most common morbidity after ostomy closure, and the rate varies widely from 0 to 40% [1, 9, 10]. Compared to conventional primary closure, several procedures have been adopted to reduce the risk of surgical site infection [11–13]. The use of a purse-string suture for closure of surgical skin defects is found with low-infection rate [14, 15]. However, colostomy is not the focus of these studies, which has different risks and wound infection rates when compared with ileostomy [16].

Mobilization of the colostomy is a critical step in takedown. Inadequate traction and stool spillage may prolong the operative time and increase the wound infection rate. Preceding temporary closure of colostomy with silk suture enhances traction and changes the “contaminated” wound to “clean- contaminated” before the colostomy is mobilized. In this study, we compared two groups of patients receiving closure or not to determine the effects on postoperative outcomes.

## Materials and methods

### Patients

The study was approved by the Chang Gung Medial Foundation Institutional Review Board. Patients who underwent loop colostomy takedown were included in the study. Clinicopathological features of each patient were recorded, which included age, gender, body mass index (BMI), comorbidities, interval from creation of loop colostomy to closure, preoperative hemoglobin and hematocrit data, and American Society of Anesthesiologists (ASA).

### Operative technique

**Fig. 1:**
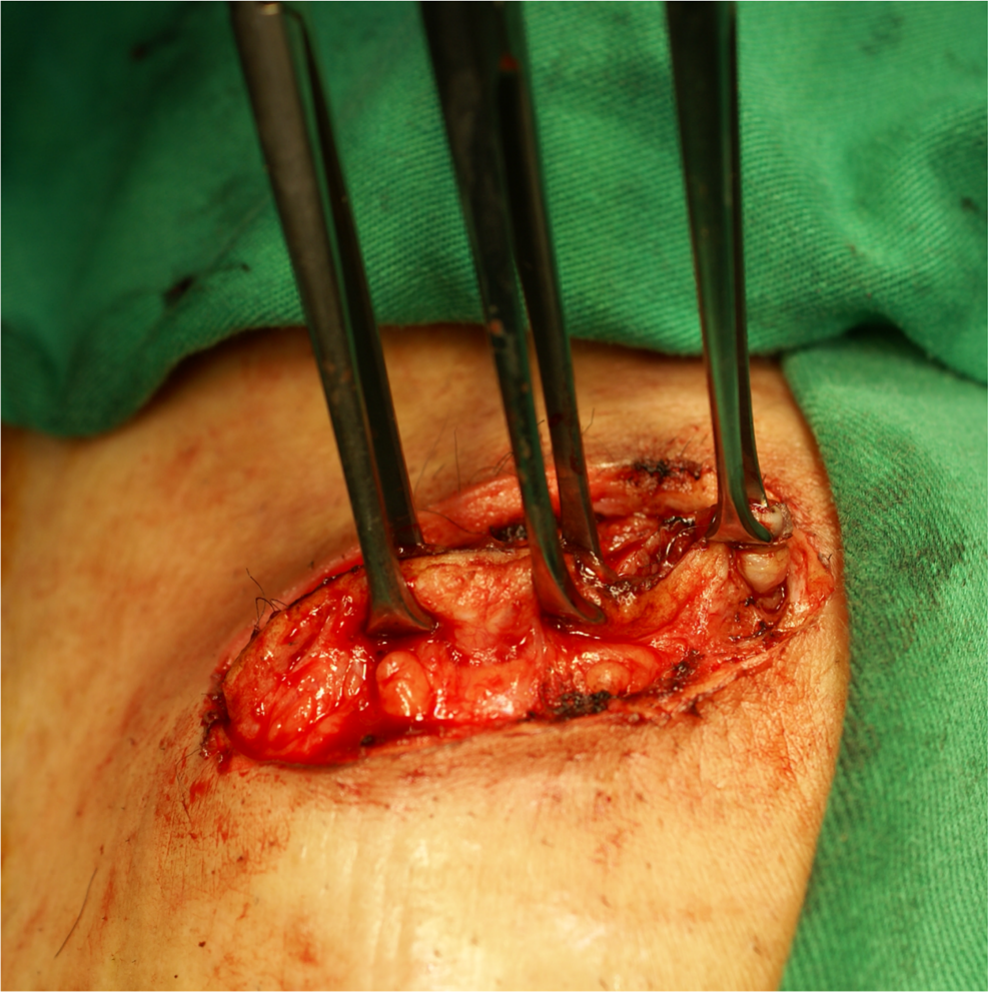
Closure of colostomy with Allis clamp

**Fig. 2:**
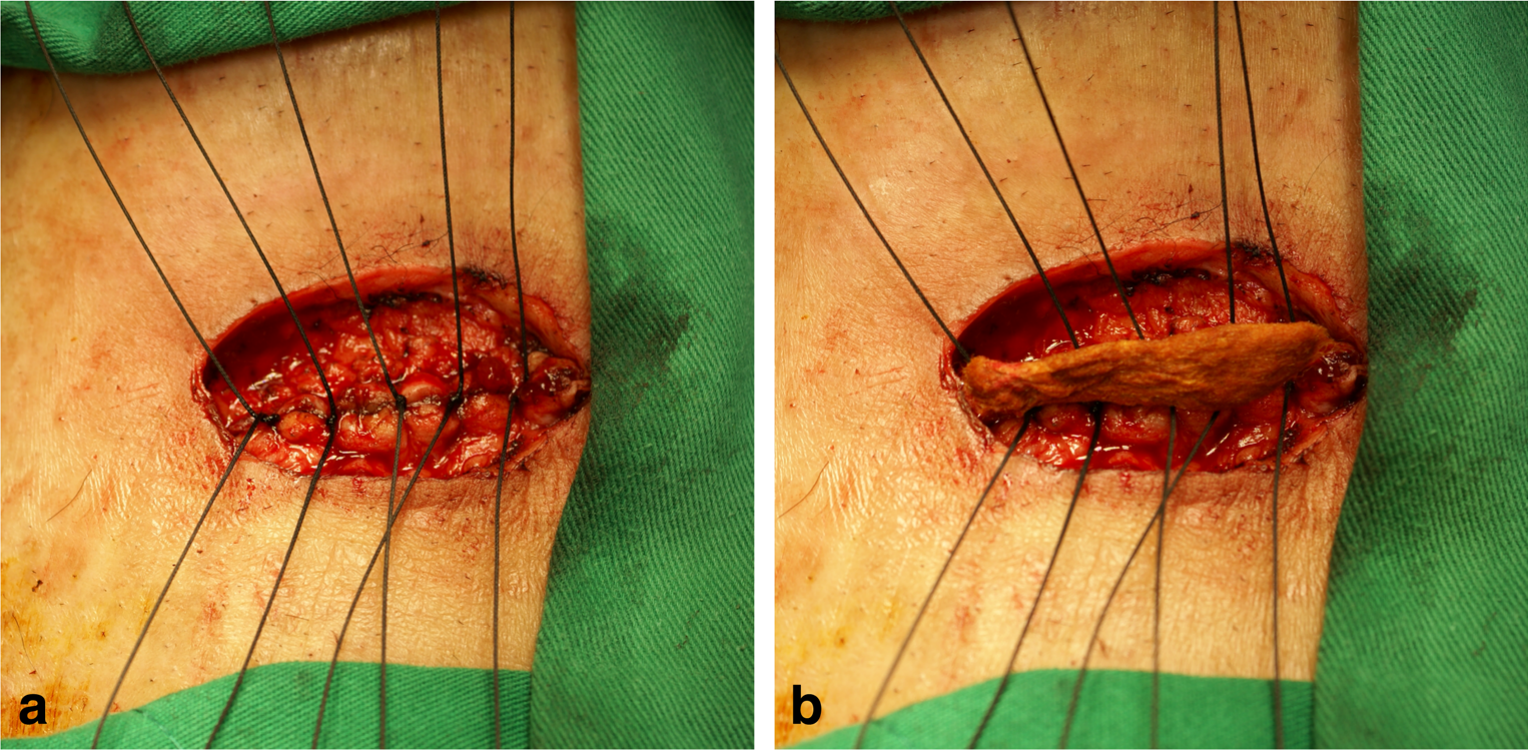
Closure of colostomy with silk suture (**a**) and povidone iodine gauze on the surface of ostomy (**b**)

**Fig. 3:**
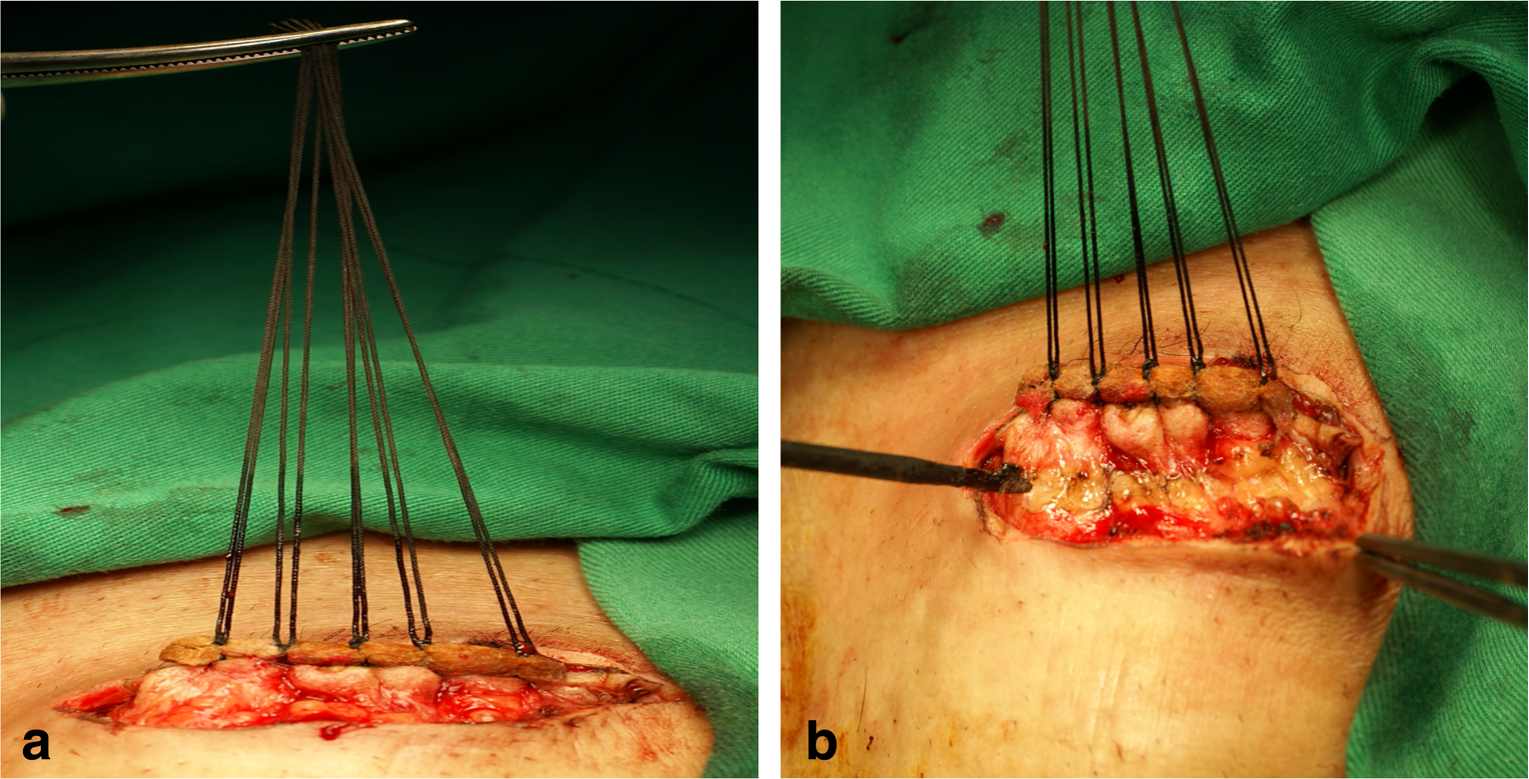
Traction of silk suture (**a**) and further dissection (**b**)

Preoperative bowel prepares included clear liquid diet, preoperative prophylactic antibiotics (first-generation cephalosporin and fasigyn), and three-way irrigation. After incision of peristomal skin, the surgeon decided which procedure the patients were going to receive before mobilizing the colostomy. Allis tissue forceps were used to clamp the ostomy in the Clamp group (Fig. 1). In the Suture group, temporary closure of the ostomy with silk suture was performed (Fig. 2a). Povidone iodine gauze was tied on the surface of ostomy for sterilization and avoidance of stool leak (Fig. 2b). Traction provided by suture was used for further dissection (Fig. 3a and b). Mobilization, excision of ostomy edge with skin, anastomosis with absorbable suture, repair of the rectus sheath, and primary closure of wound skin were same in both groups.

### Outcome measurements

Operative time and wound infection rate were primary end-points. Operative time was measured from takedown start to takedown finish [17]. Wound infection was identified by the review of progress notes, e.g., clinical findings of localized cellulitis, need for antibiotic treatment, and/or opening of the wound [12, 18].

### Statistical analyses

Fisher’s exact test was used to determine the association between groups and categorical variables. The Mann-Whitney *U* test and an unpaired *t* test were computed for continuous variables. Multivariate analysis was adjusted with significant preoperative demographic and clinical factors. Multiple regression analysis and multivariate logistic regression were used to demonstrate the associations with operative time and wound infection, respectively. A *p* value of < 0.05 was considered significant. All analyses were performed using Prism 7 and SPSS version 24.

## Results

A total of 110 patients who underwent takedown of loop colostomy were included in this study. Hand-sewn anastomosis was directly performed, and no colectomy was needed for every patient. There was no significant difference in clinical characteristics between Clamp and Suture groups (Table 1).

**Table 1:** Clinical characteristic of patients who received closure of colostomy stratified by different procedures

Characteristics	Clamp (*n* = 50)	Suture (*n* = 60)	*P* value
Age, year	62 (83–29)	62 (85–20)	0.57
Gender, male/female	33/17	41/19	0.84
Body mass index	23.6 (16.9–29.2)	22.4 (18.5–29.3)	0.34
Diabetes mellitus	9	6	0.27
Liver cirrhosis	5	1	0.09
Uremia	1	0	0.45
Interval to closure, day	142.5 (89–386)	136.5 (91–846)	0.66
Hemoglobin	13 (16.7–9.4)	12.9 (16.6–6.7)	0.42
Hematocrit	39.1 (47.7–29.6)	38.3 (47.9–21.6)	0.24
ASA (> 3)	10	15	0.64

*ASA* American Society of Anesthesiologists

Values of continuous variables: median (range)

Operative time and wound infection rate were compared for the two groups. The study results were shown in Table 2, which demonstrated that mean operative times in Clamp and Suture groups were 69 and 57 min, respectively, indicating significant (*p* = 0.003) (Table 2). Multiple regression analyses results and patients’ clinical characteristics were also shown in Table 2, revealing that increased operative time was significantly noted for patients in the Clamp group (*B* = 8.5, *p* = 0.01).

**Table 2:** Simple and multiple regression analysis for operative time

Variables	Simple		Multiple	
	*B* (95% C.I.)	*P* value	*B* (95% C.I.)	*P* value
Age	− 0.14 (− 0.41–0.13)	0.31	–	–
Gender, male	5.38 (2.87–13.63)	0.19	–	–
Body mass index	1.17 (−0.13–2.47)	0.07	–	–
Diabetes mellitus	15.55 (4.58–26.53)	0.006	9.21 (1.23–19.66)	0.08
Liver cirrhosis	36.07 (20.3–51.84)	< 0.001	29.09 (13.1–45.04)	< 0.001
Uremia	− 12.6(− 53.7–28.3)	0.54	–	–
Interval to closure	− 0.01 (− 0.02–0.04)	0.56	–	–
Hemoglobin	0.88 (− 1.36–3.13)	0.43	–	–
Hematocrit	0.55 (− 0.27–1.39)	0.18	–	–
ASA (> 3)	2.66 (− 5.98–11.39)	0.54	–	–
Suture group	− 11.7 (− 19.2–− 4.2)	0.003	− 8.5 (− 15.6–− 1.48)	0.01

*B* coefficient, *ASA* American Society of Anesthesiologists

Wound infection based on clinical findings occurred in eight patients in the Clamp group and two in the Suture group. The wound infection rate was significantly lower in the Suture group than in the Clamp group (3 vs 16%, *p* = 0.03) (Table 3). *Escherichia coli* were found in all patients with a positive culture. After adjusting for other clinical covariates, a significantly lower wound infection rate was found for patients in the Suture group (odds ratio = 0.18, *p* = 0.04) (Table 3). Patients in both groups had the same average length of hospital stay (7 days). The median length of hospital stay in uninfected patients was significantly shorter than that of the infection patients (6.5 vs. 12.6 days, *p* < 0.001). The average of length of wound infection treatment was 1 week. No anastomotic leakage was noted in any patients.

**Table 3:** Univariate and multivariate logistic regression analysis for postoperative wound infection

Variables	Univariate		Multivariate	
	OR (95% C.I.)	*P* value	OR (95% C.I.)	*P* value
Age	1.07 (1–1.14)	0.03	1.06 (1–1.13)	0.04
Gender, male	1.14 (0.27–4.73)	0.84	–	–
Body mass index	1 (0.8–1.24)	0.98	–	–
Diabetes mellitus	0.683 (0.08–5.81)	0.72	–	–
Liver cirrhosis	0	0.99	–	–
Uremia	0	1	–	–
Interval to closure	0.99 (0.98–1)	0.39	–	–
Hemoglobin	0.98 (0.67–1.43)	0.94	–	–
Hematocrit	1 (0.88–1.17)	0.79	–	–
ASA (> 3)	2.66 (− 5.98–11.39)	0.54	–	–
Suture group	0.18 (0.03–0.89)	0.03	0.18 (0.03–0.95)	0.04

*OR* odds ratio, *ASA* American Society of Anesthesiologists

## Discussion

Temporary closure of a colostomy with silk suture shortened the operative time and lowered the wound infection rate. We adopted the preceding procedure to improve the postoperative outcome of takedown for loop colostomy.

The operative time for takedown of loop colostomy varied. The mean operative time reported by Edwards et al. was 48 min [6], and the time reported by Law et al. was 51 min [7] in randomized clinical trials. In some retrospective studies, the median operative time ranged from 72.6 to 116 min [1, 11]. In two randomized trials, the average operative time was 127.2 and 116.4 min, respectively, for loop ileostomy and colostomy [14, 15]. It would be unfair to conclude that a takedown procedure was better based on shorter operative time alone, as there are many other confounding factors. However, the results of multivariate analyses demonstrated that the operative time for temporary closure of colostomy was significantly decreased in our study. This might be attributed to the silk we used for suture of the colostomy. The suture silk provided us adequate and omnidirectional traction to separate and dissect the adhesive ostomy from the abdominal wall easily and quickly. However, the traction was deficient in the Clamp group. The difficulty to hold the all Allis and unequal distribution of traction force caused the edge damage of the colostomy, wound contamination and the prolonged operative time. Increased operative time is associated with higher anesthesia cost [19] and more postoperative complications [20, 21]. Further clinical trials should be designed to confirm the benefit of the surgeries with a shorter operative time.

The wound infection rate in ostomy takedown is believed to be associated with the techniques of wound closure [13, 22]. Purse-string closure was associated with fewer wound infections than primary closure in randomized controlled trials [23]. The wound infection rates ranged from 0 to 16%. However, the studies included the patients with an ileostomy [24, 25], which had a lower risk of wound infection than a colostomy [1, 16, 26]. Temporary suture closure preceding mobilization of the colostomy avoided stool spillage and altered the wound classification from “contaminated” to “clean-contaminated,” which led to a lower wound infection rate (3%) in our study, even although conventionally primary closure was used.

No significant difference between the two groups for the average length of hospital stay was found in our study. This finding was similar to the results of different closure techniques published previously [23, 27]. The reasons why lower wound infection rate did not seem to impact the length of hospital stay might be that the wound infection rate was too little to cause an obvious difference in hospital stay between the groups. However, our study showed that hospital stay was predominantly prolonged for patients with wound infection, which was consistent with the finding in prior reports [28, 29]. Any procedure designed to reduce wound infection rates should be applied to avoid lengthened hospital stay and the expenses derived.

Li et al. reported that increased BMI was a predictor of surgical site infection after a takedown procedure [22]. Mirbagheri et al. also reported that morbid obesity was significantly associated with increased risk of infection [10]. However, there was no significant difference in BMI among those with and without wound infection in our analyses. This might be attributed to the relatively low BMI our patients had, though a trend toward higher surgical site infection rates in patients with a higher BMI was found in previously published data (*p* = 0.051) [30]. Whether BMI predicts for surgical site infection after stoma reversal is still widely debated [15].

Patients with liver cirrhosis were found to have lengthened operative time in our study. Higher surgical mortality and morbidity are noted in cirrhotic patients receiving elective surgery [31] or colorectal cancer operation [32]. However, the postoperative outcomes of colostomy takedown in the patients with liver cirrhosis are scantly published. Nevertheless, bleeding stomal varices are reported to be associated with liver cirrhosis [33, 34]. Lengthened operative time in cirrhotic patients might be attributed to the time need to control the bleeding. Further studies are warranted to identify the role of liver cirrhosis in closure of colostomy.

There are several limitations to this study. First, this was not a randomized controlled trial. Second, the results for wound healing times were not shown because the exact days were not available, but the wound healing time of most patients was less than 2 weeks. Finally, the limitation in long-term follow-up made us unable to investigate the incidence and risk factors of incision hernia in temporary ostomy wound. It is still controversy if ostomy wound infection is associated significantly with incision hernia [35–37].

## Conclusions

In our study, shorter operative time and decreased wound infection rate were achieved with the use of the preceding suture closure. Further prospective randomized controlled trials are needed to demonstrate the benefit of this procedure in colostomy takedown.
